# Practical Observations on the Nature and Treatment of Nervous Diseases

**Published:** 1836-01-01

**Authors:** 


Practical Observations on the Nature and Treatment of Nervous Diseases; with Remarks on the Efficacy of Strychnine in the more obstinate Cases. By *Geo. Russell Mart*, M.R.C.S. Octavo, pp. 185. Churchill, London, Aug. 1835.

Mr. Mart, who is a practical and observant surgeon of this metropolis, informs us that the object of the work is twofold—first, to detail the different diagnostic symptoms which mark the rise and progress of palsy, amaurotic blindness, nervous indigestion, tic douloureux, and neuralgia—and, secondly, to demonstrate the complicated nature of these diseases, shewing that they are frequently dependent on the state of the blood, and on general constitutional disorder. Hence the necessity of general remedies, as well as local. The chief portion of new matter, however, in this work, consists of numerous cases of chronic, and usually incurable diseases, treated by strychnine—a remedy not yet in extensive employment, nor sufficiently ascertained as to its remedial agency. The volume contains four chapters—the first of which is on—

### Paralytic Diseases.

After some general remarks on the ignorance of the ancients, and the discoveries of the moderns respecting the pathology of palsy, Mr. M. informs us that he has been in the habit of prescribing strychnine for some years, in the chronic forms of the malady in question, and assures us that it is a remedy of great power and utility. We shall advert to a few of the cases in each chapter, abbreviating them in most instances.

*Case* 1. Mr. Wyatt, King Street, Soho, had paralysis of the right side including the muscles of the face and tongue. He had rather a fatuous look. The paralytic affection had commenced three years previously, and various remedies had been tried in vain. One-sixteenth of a grain of strychnine was first prescribed, thrice a-day. After two days, the dose was increased to an eighth—and afterwards gradually augmented to a third of a grain. In ten days, some slight twitchings about the face were remarked, and they soon extended to the arm and leg. They increased even to annoyance. At the end of three weeks, he had more power in his arm, and could stand on his legs. By the fifth week he could walk, with assistance, about the apartment. The strychnine was continued, in doses of half a grain in the 24 hours. The expression of the countenance altered, from silliness to archness, and, at the expiration of seven weeks, he walked without assistance. In four months, he was able to resume his usual avocations. This was in the end of 1829 and beginning of 1830. In July, 1832, Mr. Wyatt was seized with apoplexy, from which he narrowly escaped. It was followed by paralysis of the right side. The use of the limb, however, returned in four months, so that he was able to walk to town—a distance of three miles from the Edgeware Road, where he now resides. He is apparently in good health, and can take considerable exercise. He used the strychnine for the secondary paralysis.

*Case* 2. The patient was Thomas Fryer, placed under Mr. Mart's care by Viscount Gage. His age was 34, of full temperament, muscular and plethoric. For four years, he had little use of his left leg, and still less of the corresponding arm. His sight was materially affected. The case was aggravated by epileptic seizures, occurring at intervals of ten, fourteen, or 21 days. After some aperient medicine, the strychnine, in doses of an eighth of a grain twice a-day, was administered—the quantity being gradually increased to a quarter of a grain ter die, when shaking fits were complained of in the paralyzed parts. After two months of this treatment, an attack of epilepsy interrupted the medicine for a few days, when it was resumed, and continued for several months (with epileptic interruptions).

"The result of this treatment was, that in two months he could walk a distance of half a mile, and the palsied arm acquired strength. After four months he was able to employ himself in French polishing, and in manufacturing light pieces of cabinet-work.

The fits of epilepsy returned at longer intervals of time, and abated much in severity; ultimately they dwindled to peculiar and rather painful sensations in the leg, such as usually are denominated premonitory symptoms of epilepsy.

Fryer now walks to various parts of the town, carries home his work, and when fully employed can maintain his family in a comfortable manner." 16.

Several other cases of hemiplegia are detailed by Mr. Mart; but the above are sufficient specimens.

### PARAPLEGIA.

The cause of this severe malady is sometimes in the head, though more frequently in the spinal marrow. The following extract will shew that Mr. Mart is an attentive observer of morbid phenomena.

"The symptoms of this form of Palsy are as variable as the causes. With some, the disease commences with slight occasional pains, referable to a part of the hip, thigh or leg, and frequently behind the protuberance at the head of the thigh-bone. Pain is not always present; fatigue frequently induces it, and rest relieves. Weakness and pain now manifest themselves in the muscles at the lower part of the back, or in various parts of the legs. The sensation of a cord tied round the calf of the leg is a common symptom. At times the patient cannot walk, or even stand erect, without a paroxysm of pain following, which compels him to assume a recumbent position, as the only mode of obtaining partial relief. Locomotion is performed with difficulty; the legs threaten to give way, and bend under the patient at every step.

An additional support is now required in moving about, as crutches, or a stick. As the malady increases, an occasional involuntary discharge of urine takes place, and incontinence of the lower bowels ensues. Sometimes, on the contrary, the greatest difficulty prevails in invoking the natural action of the bowels, and various domestic instruments are had recourse to, with very little avail.

After an interval of months or years, the legs become incapable of carrying the body beyond a few yards, and sometimes the erect position cannot be maintained while sitting, the power of moving the legs may remain, and sensation be unaffected. Generally a wasting of the paralysed limb is apparent, while sensation may be morbidly increased.

On sudden and rapid changes of weather, the patient is attacked with painful *neuralgic* twitches, resembling those of Tic Douloureux.

There is a peculiarity in the walk of these patients. They first press on the heels, and the toes waver, turning inwards or outwards. They straddle or bow their legs to obtain footing. Patients live in this state an indefinite period." 34.

*Case* 3. Thomas Linton had been two years in the Racoon Hospital-ship (Portsmouth Harbour), before Mr. Mart's appointment to that ship. He was tall, thin, and sallow—very intelligent. He had not quitted his bed for 20 months, except when carried on deck for fresh air. The cause of the paraplegia was a blow on the loins by a fragment of stone. The symptoms were slight at the time; but afterwards a sense of numbness extended along the thigh, with a feeling as if strings were tied round the legs. These symptoms increased, and, in three weeks, he was deprived of power in the legs, and unable to stand. Various means had been tried, but without any good effect.

"The treatment was commenced by administering some blue-pill, and doses of purgative medicines; the state of the secretions required previous treatment. Afterwards, one-eighth of a grain of strychnine was ordered in the form of a pill twice a-day, and a dose of a mixture containing *diluted sulphuric acid* taken at the same time. On the second day the pill was repeated three times, and the strychnine was gradually increased to a grain in twenty-four hours. When the treatment had been continued a month, no amendment occurred; but about this time, a blister was applied over the part where the blow was received.. On removal of the skin, one-quarter of a grain of strychnine was applied twice a day. The blister was dressed daily in this manner till it healed, when another was applied in the vicinity, which was dressed with half a grain of strychnine, sprinkled over the denuded surface twice a-day, and also administered internally in pills, containing a quarter of a grain, four times a day. Six weeks from the commencement, the patient began to improve; convulsive jerks were felt in the legs, and a sense of pricking and other odd sensations were experienced: the toes would move involuntarily. An erect posture had been attained several days; the patient sat in a chair, and moved the legs in every direction. He continued to improve in various degrees; but the amendment was always more rapid after blistering. This treatment was continued four months, when he was so far recovered as to be discharged from the Hospital Ship, and had light work assigned him. Finally the cure became perfect, and he performed the duties of an able-bodied man." 37.

*Case* 4. This was a medical gentleman, Mr. Martindale, who became paraplegiac on his voyage home from India. The strychnine was proposed, but he was persuaded to try the air of Wales for a few months. This made little or no improvement in the paralytic complaint, and, on his return to London, he complied with Mr. Mart's prescriptions. He began by taking an eighth of a grain of strychnine thrice a-day, gradually increasing the dose. In the course of two months, he walked without support; and his bowels, which had been very irregular, assumed their natural action.

*Case* 5. This case being short, we shall give it in the words of the author.

"In the year 1832, the daughter of an officer in the army, residing at 25, Arundel Street, came under treatment. This lady, in 1829, complained of a heavy, dull pain at the back of the neck, sometimes approaching to the front. When the pain was momentarily increased, she was obliged to grasp her neck with both hands. Difficulty of swallowing at times was felt, when pain extended to the muscles and parts of the chest, and about the shoulders. These were ascribed to rheumatism. The tips of the fingers felt benumbed, and she experienced considerable annoyance from the state of her bowels, and the irregularity of the monthly indispositions. The toes were affected with numbness, and the joints of the lower extremities were stiff and painful. Subsequently, the loss of motion increased, and the power of walking and standing was destroyed. When the treatment commenced, the legs were emaciated, and the muscles soft and flabby. The expulsion of fæces and urine was attended with difficulty. Large patches of inflammation, threatening to produce sloughing ulcers, were apparent on the upper and posterior parts of the thighs. For three months, strychnine pills were administered. The constitution being delicate and much shattered, only one-twelfth part of a grain was first ordered to be given night and morning, and afterwards three times a-day. This was increased to one-eighth of a grain, the greatest dose administered in this case. Five small blisters were applied to the loins during the treatment, and one quarter of a grain of strychnine used twice a-day, while the blistered surface was in a condition for its absorption. At the end of the treatment the patient could stand, and, with assistance, shuffle the feet along the floor. Quinine pills were now ordered, and continued for a month.

The improvement was slow; twelve months elapsed before she could walk abroad; the legs had recovered their former plumpness, and the constitution much of its original vigour." 41.

Mr. Mart next adverts to the paralysis agitans, or shaking palsy—a peculiar disease, and which we have found, contrary to Mr. Mart's experience, to be a most obstinate, and generally incurable disease. We were, therefore, a little surprised to read the following paragraph, at page 44 of the work. "This disease does not usually produce derangement of the general health; and patients sometimes live to an old age, or die of other complaints." "The strychnine is remarkably efficacious in nearly all cases of 'SHAKING *palsy*.' We shall extract the only case that is given by Mr. Mart, "from *numerous* cases in the author's note-book"—a statement that also surprises us, since a long, and not very limited, experience has not furnished us with more than half a dozen instances of unequivocal paralysis agitans. We have never seen recovery in any one case.

*Case* 6. "Miss C—k, aged 18, residing at a school in Woolwich, was observed, when about 14 years of age, to draw up her feet quickly and suddenly in walking; she complained of numbness in her fingers, and could not use a needle; the fingers and thumbs of both arms began to move involuntarily, attended with spasmodic twitches of the muscles; the legs, about the same time, were affected with similar agitations. Her gait was unsteady. She was able to project her legs, but in no other way could control them. The head was affected by spasmodic movements, being sometimes tossed disdainfully backwards, or on either side.

This young lady had the best advice, and, in the early stage of the disease, was properly treated. On the malady degenerating into a chronic type, her friends considered it was useless to make further attempts to procure relief, as no remedy appeared to be of service.

When first seen by the author, the limbs were agitated as she sat in a chair, and when desired to walk to a particular part of the room, it became a matter of doubt if an opposite direction was not about to be taken, or one to the right or left; in short, she proceeded in a zig-zag way. Her health was tolerably good; no deformity was present; her stature was of the middle height, but her person was rather thin. The treatment was continued eleven weeks, commencing the strychnine with one-twelfth of a grain, increased to one-eighth of a grain three times a-day in the form of a pill. At intervals blisters were applied, and the spine rubbed with strychnine, dissolved in spirit, after the prescription of M. Majendie.

The symptoms finally yielded to the efficacy of the remedy, and her constitution improved in strength. When her feelings are affected by circumstances not of an ordinary nature, the limbs tremble, and the fingers move, but these symptoms pass off on recovering herself." 46.

We think we may confidently affirm that the foregoing case was *not* one of shaking palsy, but of CHOREA. And this mistake will account for the numerous cases of the disease which Mr. Mart has entered in his note-book. The candid manner, however, in which Mr. M. has stated the symptoms, absolves him from all intention to deceive his brethren. If authors were thus to act, the science of medicine would not be blotted so often by "false facts" as it long has been!

### AMAUROSIS.

In this disease, too often found incurable, "strychnine has been highly beneficial in the hands of the author."

"At the onset of this malady some constitutional symptoms generally are present, such as derangement of the digestive organs, torpidity of the liver, and pains about the front of the head: sometimes indeed symptoms referable to palsy, come on at distant parts of the body; transient flushes of the face occur, and patients are assailed with fanciful sounds, as buzzing noises, and the dropping of water; or alarming sensations are created by the forcible pulsation of the internal vessels of the neck and head. Among the primary symptoms of impaired vision, are the appearances of insects, cobwebs, or a piece of gauze appended between the affected eyes, and the object viewed; these observations increase in magnitude, and become more opaque. Sometimes the failure of vision is only occasional, recurring at longer or shorter intervals.

Zones of bright light appear to surround objects, and flames of candles or lamps assume every position but the real one. Rays of light flit about the eyes, scintillations verge out in every direction, or yellow sparks pass across at every turn of the orbit, occasionally becoming vivid or forked like lightning." 53.

Five cases are detailed by Mr. Mart. We shall briefly notice some of them.

*Case* 7. Mr. W., a stockbroker, had suffered several years from defects of vision. "At first he became unable to read by candlelight, afterwards he had pain at the bottom of the orbit, while the external parts of the eyes were tender and irritable, and when pressed, however gently, pain was produced; he had a dull pain in the forehead, generally aggravated at night, and there were frequent involuntary movements of the eyelids. He saw best at noon-day in the sun, or in a room brilliantly illuminated. He transacted all business in which a pen was required as expeditiously as possible, for whenever the eyes were employed, especially while writing, uneasy sensations invariably occurred." When the treatment was commenced he wrote in large characters, and he could not recognize the features of a person at the distance of three yards. The pupils were generally dilated. Strychnine was given night and morning, in doses of a tenth of a grain. Small blisters were applied over the eye-brows, and dressed twice a-day with a quarter of a grain of strychnine. This treatment was continued seven weeks. The patient was very irritable, and would not continue the blisters. A spirituous solution of the strychnine was therefore applied to the neighbourhood of the eyes for four weeks. In the course of a fortnight candlelight ceased to produce pain, and the power of vision was improved. He did not, however, completely recover.

*Case* 8. Fryer, (Case 2, already noticed) had amaurotic blindness of both eyes. "When in a room he could only distinguish the aperture through which the light entered. He knew no one by sight," nor could he distinguish a male from a female by their dress in the streets. This state of vision followed a severe attack of apoplexy occurring subsequently to the paralysis before described. He had epileptic fits every month. He was under treatment for eight months, and was twelve times blistered about the outer angle of the eyes and over the eye-brows. The restoration of vision was very gradual. Many weeks elapsed before the slightest improvement took place. When last examined he detected a fly creeping on the window. His vision was always deteriorated previous to an attack of epilepsy. These fits have now disappeared, and the sight continued to improve long after the treatment was left off.

*Case* 9. Mr. D. residing in the New Road, had been afflicted with amaurotic blindness many years. The deprivation was sudden, and both eyes were affected. "The patient can scarcely distinguish the outlines of a human form, and requires a strong light even to perceive a white object on a dark ground." He has difficulty in standing or walking at times. "During these attacks of nervous debility in the lower extremities, and whenever great atmospherical vicissitudes occurred, he was seized by neuralgic pains at various parts, frequently preventing sleep at night, and causing much disquietude through the day."

The principal seats of pain were the legs and arms. This patient had been seen and treated by some of the most eminent medical practitioners. The strychnine was employed for three months, internally and externally, together with blisters. The result was decided benefit—so that the patient could walk round the Regent's Park. It is curious that, one day during dinner, the sight became almost perfect; but soon deteriorated, and has since remained so. Two or three other cases are related; but we cannot afford space for their details here.

### CHAP. II.—NERVOUS INDIGESTION.

This chapter is not susceptible of analysis, though it evinces considerable powers of observation in the author. We confess that it is the least satisfactory chapter in the work. The following passage will serve to explain this.

"Nervous affections of the stomach, and of other parts of the system, are often subdued by a well-directed course of medicines; but sometimes they resist every mode of treatment, and it is in such instances that strychnine should be administered.

This remedy, however, will not always succeed in eradicating the malady. The difficulty in some instances of removing the causes of nervous disorders has frequently prevented the strychnine from affording more than partial relief. When the causes were as active as at the commencement of the disease, no benefit has accrued." 101.

The third Chapter is on—

### Tic Douloureux.

This is a disease of modern recognizance, though, probably, it has existed time immemorial. Fothergill, in 1766, gave the first description of it, and our continental neighbours, since that period, investigated its nature and causes with more zeal than success. Its etiology and pathology are indeed involved in *impenetrated,* if not *impenetrable* obscurity!

" The attention of the medical profession has lately been directed to the production of this disease by the growth of bony particles within the head, operating through the medium of the brain. Although these and similar causes are always present, yet the pain is generally of an intermittent character. The same obtains in other nervous diseases, as has been fully demonstrated in the results of dissections of persons whose brains were penetrated by points of bone growing from the internal surface of the bones of the head, but who during life had *periodical* head-aches, or epileptic seizures.

Upon a more extended view of the subject, of its origin, and its primary seat, the probability is, that the source of irritation is more frequently in a portion of the brain; sometimes in those portions from which the nerves of the face have their immediate origin,—and sometimes at the roots of the nerves themselves. That the seat of the disease is not often in the sentient extremities of the nerves which are distributed over those parts of the face where pain is experienced, may be clearly inferred by the failure of ordinary and external means of obtaining relief, even including the operation of dividing the nerves." 108.

Mr. Mart thinks, and probably with reason, that the disease consists more frequently in functional *disorder* than in any cognizable *disease* of structure in the parts affected—or may be sympathetic of disease, or even disorder, in a distant part. Hence it is, that medicines directed to the constitution generally, are more beneficial than local applications. Mr. Mart has found strychnine very useful in this painful malady. He has frequently found the blood of patients labouring under neuralgia to be *buffy,* and he seems inclined to connect a diseased condition of the blood with tic douloureux. We shall now glance at one or two of the cases.

*Case* 10. A young officer had been exposed to wet, and suffered from what was considered to be rheumatism of the face for several weeks. The pain remitted in the night. The extraction of a molaris relieved him for three or four months. But in the succeeding Autumn the paroxysms of pain became almost insupportable, and he had been the victim of intense suffering. In this condition he applied to Mr. Mart. The principal seat of the pain was now the left side of the upper jaw, extending to the nose, corner of the eye and forehead. The upper lip and front teeth were also affected. The gums were red, swollen, painful, and spongy. The general health appeared good. The gums were freely scarified, and it was found that some portions of the sockets were denuded.

" The patient was directed to apply daily two small leeches to the gums of the upper jaw. After six applications the symptoms were palliated, the attacks diminished in intensity, and the remissions between the paroxysms were longer. He continued thus improving for twenty days, when the disease became stationary. The pain being now more obtuse, the cheek could be pressed with impunity. The side of the face was rubbed with a spirituous solution of strychnine, (one grain and a quarter being used at each application), three times a day. A sense of heat and tingling was felt in the skin. The attacks gradually lessened, and after persisting in the external use of the remedy seven weeks, the patient found himself free of every uneasy sensation. Three months afterwards the patient informed the author, that there was a disposition in the disease to return, and of his own accord he had used the strychnine fourteen days. More than two years have now elapsed without a recurrence of the malady." 126.

*Case* 11. A relation of the Marquess of Anglesea had been afflicted for many years with tic douloureux, the attacks continuing for indefinite periods. The pain was chiefly in the right side of the face. One or two suspected teeth had been extracted. He had occasional fulness in the head. Medical assistance, of the first quality, had been procured, yet to no purpose. There appeared to be no derangement of the general health, excepting that the circulation was rather excited. Some blood was taken from the arm, and presented the buffy coat. Then eighteen ounces were abstracted, and evinced the same quality. The detraction was repeated in a couple of days, and salines were exhibited. The facial pain became sensibly diminished. Exposure to the inclemency of the weather in a journey to Brighton, produced a relapse, and on taking blood, it was not found buffy. The strychnine was now exhibited, and applied externally. In a month the pain was gone. The patient then went to the Continent, and the sequel of the case is unknown.

After stating some other cases, Mr. Mart concludes with this sensible observation.

" Strychnine must not be considered as a specific. It will not succeed in every case. Generally, however, its effects are beneficial, and considerable confidence may be placed in its virtues. It is not to be used to the exclusion of other remedies ; but when cases occur which have resisted the usual modes of treatment, a trial of this remedy will generally be attended with mitigation of suffering, often with permanent relief." 148.

### Neuralgia.

This class of painful complaints has excited great attention for many years past. Our author is inclined to give neuralgia a very wide range—wider even than that which has been ascribed to it by a late talented writer —Dr. M'Culloch.*

" When the facial nerves are affected, or when the pain is confined to the course of the nerves in the upper or lower extremities, little difficulty arises in

* We were sincerely grieved to learn the melancholy fate of Dr. M'Culloch. It appears that, in jumping out of a chaise, he received so bad a fracture of one of his legs that amputation was rendered necessary, and that he died soon after the operation! He was a man of great, though eccentric talent—and his loss is deeply to be deplored by science in general, and medical science in particular!

detecting the disease; but very frequently symptoms of an anomalous description appear, and assume various characters, the origin of which is in the majority of cases exceedingly obscure. Many of those indescribable and tedious complaints which principally affect persons in the highest ranks of life, especially females, are often connected with functional disorder of the nervous system. They often manifest themselves by weakness in portions of the body, often in particular parts of the legs and arms, or of the back. Some of the symptoms are referable to the internal organs. Sometimes the urinary apparatus or bowels are affected. Occasionally disorder of the digestive and pulmonary organs appears under the various forms of asthma, palpitation, spasms, and head-ache. 'There are few physicians who may not on reviewing many cases which have occurred to them of anomalous pains in different parts of the body, so as to counterfeit *gouty, bilious,* and other internal affections of the stomach and bowels, perceive some analogy between them and the complaints here pointed out.'" 151.

We need not detain our readers with an enumeration of the symptoms of neuralgia, as detailed by our author. Several cases, some of them well-marked, are related by Mr. Mart, in which strychnine appears to have been very advantageous. In a disease, therefore, so generally intractable as neuralgia is known to be, the remedy in question may be fairly recommended as a "dernier resort." We place more confidence in the statements of Mr. Mart, than in those who hold their heads much higher in the medical world, because we know him to be a straight-forward "HONEST MAN"—who, according to Pope, and many others, ought to be considered as—the "noblest work of God." Of all arts, sciences, or avocations, MEDICINE is that which ought, for its own sake, and the sake of humanity, most dearly to cherish HONESTY. It is a science which contains the purest ore combined with the most worthless dross! From its nature and constitution, it will be exceedingly difficult of depuration. A thousand centuries will not constitute it a pure science. It is incapable of becoming one. It must always have a vast alloy of *conjecture;* and wherever there is conjecture, there will be Charlatanism and knavery!

